# Reconstructing illusory camouflage patterns on moth wings using computer vision

**DOI:** 10.1098/rsif.2024.0757

**Published:** 2025-04-30

**Authors:** Laurent Valentin Jospin, James Wang Porter, Farid Boussaid, Mohammed Bennamoun, Jennifer L. Kelley

**Affiliations:** ^1^ École Polytechnique Fédérale de Lausanne, Lausanne, Switzerland; ^2^ Department of Electrical, Electronic and Computer Engineering, The University of Western Australia, Perth, Australia; ^3^ Department of Computer Science and Software Engineering, The University of Western Australia, Perth, Australia; ^4^ School of Biological Sciences, The University of Western Australia, Perth, Australia

**Keywords:** camouflage, depth cues, moths, shape from shading, three-dimensional reconstruction

## Abstract

Monocular depth cues, such as shading, are fundamental for resolving three-dimensional information, such as an object’s shape. Animal colour patterns may potentially exploit this mechanism of depth perception, generating false illusions for functions such as camouflage. Reconstructing the potential percept produced by false depth cues is challenging, especially for non-human, animal viewers. Here, we provide a novel step towards solving this problem, taking advantage of state-of-the-art computer vision algorithms typically used for three-dimensional scene reconstruction. We used two approaches for single-image monocular depth estimation: intrinsic image decomposition and deep learning. We first examined how these models performed using images of natural three-dimensional surfaces that moth wing patterns may mimic. We then applied these models to the wing patterns of six species of moth (Lepidoptera) with varying amounts of potential depth information. For one species, we then performed a multi-view reconstruction of the wing pattern to reveal the true (flat) wing shape. Intrinsic image decomposition, which is based on Retinex theory, was sensitive to both real depth cues and high contrast patterns, while the deep-learning models only responded to moths with strong pictorial depth cues. Both approaches reveal how the interpretation of visual cues depends not only on the information available, but also on experience with the natural world.

## 1. Introduction

The recovery of three-dimensional shape from a two-dimensional image is one of the most fundamental properties of animal visual systems. Three-dimensional scene reconstruction also forms the central tenet of computer vision science, with applications as diverse as face recognition, autonomous driving, robotics and virtual reality (VR). Animal visual systems often rely on a combination of binocular vision and monocular depth cues to recover three-dimensional information [1]. While it is known that many animals (e.g. birds [2]) have overlapping visual fields, aside from some notable exceptions [3,4], binocular vision is not usually associated with the ability to resolve depth using stereopsis [2,5]. Depth information is therefore often obtained from monocular visual cues, which include shading, occlusion, texture gradients and linear perspective [1]. The best known monocular cue is the use of shading to resolve an object’s shape [6], often referred to as 'shape from shading’ (SFS) [7].

SFS relies on the notion that, if light comes from a single source, then the brightness of a given point on the object’s surface depends on the slant of the surface normal relative to the direction of illumination [8,9]. However, SFS is problematic because multiple points on an object’s surface can give the same brightness values, despite having different surface orientations. The human visual system resolves this issue using assumptions about how light typically interacts with three-dimensional shapes [10]. For example, the visual system 'knows’

that light typically comes from overhead, thus it can make the simplifying assumption that objects that are brighter on top must be convex, while those that are darker on top must be concave [6]. Similar assumptions are applied in computer vision algorithms, such as the assumption that an object’s surface has uniform reflectance. The problem with such assumptions is that they may be incorrect, and they can potentially be exploited to misconstrue the visual information available to the viewer. Visual illusions are a powerful way of demonstrating how these assumptions can be incorrect, leading to very different interpretations of objects and visual scenes [10].

**Figure 1.**
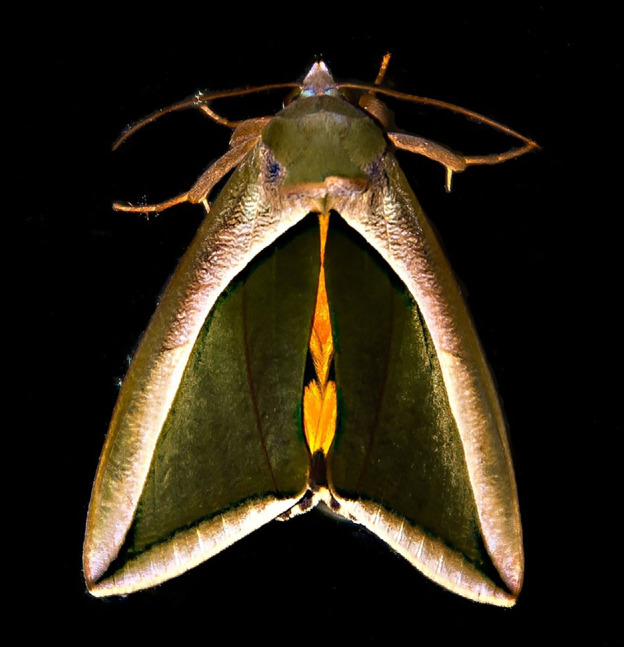
The green fruit-piercing moth, *Eudocima salaminia*, has remarkable wing patterns which, to the human observer, appear highly three-dimensional, resembling a curled leaf. Photo by Sandid via Pixabay (Content License).

Shading is used by artists, graphic designers and software engineers to generate three-dimensional scenes on a flat canvas or computer screen. Naturalists have long been aware of these principles, leading to the suggestion that animals might use their body colouration to produce a false impression of depth [11–14] to enhance camouflage, for example. One type of patterning that is proposed to serve this function is 'countershading colouration’, a common form of patterning where animals are darkest on the surface that receives the most light [12,15–17]. The combination of overhead illumination and countershading patterning has the (non-mutually exclusive) effect of reducing contrast across the body [18], enhancing background matching [19] and removing SFS cues [13,14,17,19]. Studies with wild, free-living birds and using human observers searching for three-dimensional shapes have shown that targets that are optimally counter-shaded for the illumination conditions [20,21] or the target’s orientation relative to the light source [22] are harder to detect. One study has applied computer vision to the study of countershading, showing that a convexity-based detector was fooled by images of countershaded animals [9]. An idea that has received less attention is that, in addition to removing SFS cues, animal patterning might also function to produce a false illusion of depth [14]. This strategy could potentially enhance camouflage by facilitating background matching, disrupting body surfaces and/or by mimicking inedible objects (masquerade) [23], such as leaves (figure 1).

Cott [14] suggested that animal patterns might generate 'pictorial cues’ that produce the impression of differences in surface depth or object shape where none actually exist. Evidence for this intriguing idea comes from studies [24,25] using humans searching for targets with patterns with 'enhanced edges’—colour patches that are accentuated by high contrast borders that might produce the impression of changes in surface depth [14]. Observers were slow to detect and recognize targets with enhanced edges, reporting that these patterns appeared to mimic changes in depth in the visual background [24]. However, testing whether animal patterning interferes with depth perception for non-human animal viewers is extremely challenging. Studies with wild birds as predators have found some evidence that disrupting the body’s surface enhances camouflage, but have found no evidence that pictorial depth cues can produce the same survival advantages [26,27]. Non-human predators can use SFS to resolve depth cues [28–31], and some animals can respond to depth cues in their background; for example, cuttlefish (*Sepia officinalis*) can use their remarkable colour change ability to produce pictorial cues that match natural three-dimensional objects (e.g. pebbles) [32,33]. Cuttlefish also use visual cues to change the morphology of their three-dimensional skin papillae for camouflage [34]. Thus, while many animals can resolve depth cues, it is unclear whether non-human animals are deceived by natural animal patterns that contain pictorial cues. Testing this idea would require behavioural tests of shape perception in non-human animals, which is highly challenging.

One innovative solution is to use computer vision algorithms to reconstruct animal camouflage patterns. Computer vision has previously been used to model colouration that minimizes or maximizes the probability of detection [35,36] and to model the evolution of camouflage patterns in an arms race scenerio [37], but we are not aware of any studies that have used computer vision to reconstruct the depth cues in animal patterns. The advantage of this is that there are several state-of-the-art methods for performing three-dimensional reconstructions from a single image. Furthermore, comparison of the performance of these models can provide insights into how the patterns may potentially deceive the viewer. For example, some models are based on visual theory (e.g. Retinex) and the fundamental principles of how light interacts with three-dimensional shapes. However, other models, such as those based on deep learning, adopt a different approach, learning from data (i.e. experience with three-dimensional objects in natural scenes) rather than relying on explicit assumptions about the physical world [38]. Comparing the outcome of these different types of computer vision algorithms can potentially provide a novel exploration into the ways animal colour patterns might misconstrue information about three-dimensional body shapes.

Here, we apply several approaches from computer vision to reconstruct the potential monocular depth information contained in the wing patterns of six species of moth (Lepidoptera) that vary in their patterning. Moths are ideal for this approach because the wings have a generally flat surface that often has complex patterning, including potential pictorial depth cues [23]. Most moths are nocturnal and must therefore avoid predators (such as birds) that are active during the day, when the moths are at rest. We used two approaches for single-image monocular depth estimation: intrinsic image decomposition and deep learning. Intrinsic image decomposition allowed us to estimate the relative contributions of reflectance and shading to each image, and thus to model changes in depth, while deep learning was used to determine the outcomes under a different set of priors and (mostly unknown) constraints. We first examined how both models performed when reconstructing natural three-dimensional surfaces that the moths’ patterning may be mimicking (e.g. curled leaf, bark, tree trunk). We then applied both intrinsic image decomposition and deep learning to six species of moth, which varied in their potential illusory depth cues. Finally, we conducted a multi-view three-dimensional reconstruction of one moth species *(Eudocima salaminia*, Erebidae) to demonstrate the 'true’ shape of the wing surface and the visual information obtained when the moth is viewed from multiple locations.

## 2. Methods

### 2.1. Natural three-dimensional surfaces

We captured images of natural objects and backgrounds that the moths’ colouration may be mimicking. These images were used to first compare how the computer algorithms performed with images of real three-dimensional surfaces and shapes. We used the image of a curled mandarin leaf (*Citrus reticulata*), an image of marri (*Corymbia calophylla*) bark, an image of bark damaged by a wood-boring insect, and the bark of a eucalypt photographed in woodlands (Dryandra Woodland National Park, Western Australia). Images were captured under natural illumination in RAW format using a DSLR camera (D7100; Nikon, Japan) fitted with a 60 mm macro lens (Nikkor, Japan).

### 2.2. Moth specimens

**Figure 2.**
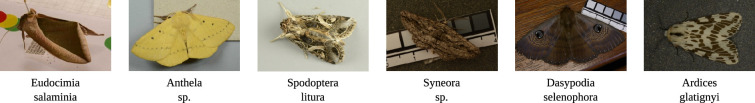
The six species of moth used in this study. From left to right: *E. salaminia* has a leaf-like like camouflage pattern with strong depth cues (to the human viewer), *Anthela* sp*.* has minimal wing patterning and no depth cues, *Spodoptera litura* has a highly textured wing pattern, *Syneora sp.* has patterns with a bark-like texture, *D. selenophora* (family Noctuidae) features prominent eyespots, while *A. glatignyi* possibly has disruptive patterning. Images: J.L.K.

We selected six species of moths from different families, which, to the human observer, appear to have varying levels of potential pictorial depth cues (figure 2). A preserved specimen of the green fruit-piercing moth, *E. salaminia* (family Erebidae), was obtained from the Australian National Insect Collection (ANIC) in Canberra. Images of an Anthelid moth, *Anthela* sp. (Anthelidae), the tobacco cutworm, *Spodoptera litura* (Noctuidae), a bark-resting moth, *Syneora* sp. (Geometridae), the southern old lady moth, *Dasypodia selenophora* (Erebidae) and the black and white tiger moth, *Ardices glatignyi* (Arctiini) were obtained from live specimens collected using a light trap (November 2016 and March 2021; Perth, Western Australia). Three other moth specimens were used for additional explorations of how the computer algorithms performed on high contrast markings (*E. cocalus,* photographed from a private collection), apsoematic markings (*Asura polyspila*, wild-caught in Queensland) and for an extra species with strong pictorial depth cues (*Uropyia meticulodina*, image obtained from Flickr). All images (other than *U. meticulodina*) were captured with the same camera and lens used to photograph natural three-dimensional surfaces (§2.1) and diffuse illumination was provided by three LED light banks (Phottix M200RGB; New York). Specimens were identified using the Atlas of Living Australia (https://www.ala.org.au).

### 2.3. Monocular depth estimation

**Figure 3.**
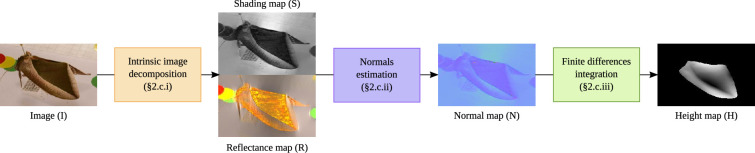
A three step pipeline for extraction of a depth map from a single image. The first step (i) is to decompose the image (I) into a shading map (S) and a reflectance map (R). The second step (ii) is to estimate the normals of the image to produce a normal map (N), while the third step (iii) is to recover the height map (H) from the normal map.

For the monocular depth estimation, we used two concurrent approaches. First, we developed a three-step pipeline (based on closed-form solutions) to extract the depth map from each image (figure 3; §§2.3.1 to 2.3.3 below). Second, we used existing deep learning-based models for monocular reconstruction (presented in §5) applied to the same moth images.

In general, the depth predicted by most monocular depth estimation methods is considered an implied or relative depth estimate (i.e. not related to physical distances) rather than an absolute depth estimate, due to the lack of an absolute reference [39]. This is adequate for our estimations as we are interested in relative depth rather than aiming to reconstruct a physical object.

#### 2.3.1. Closed-form solutions for intrinsic image decomposition

The problem of inferring the three-dimensional shape of an object from a single image is severely under-constrained. Most methods assume the underlying object has uniform reflectance R, so that any change in the image intensity I is caused by the amount of light received by that pixel, which we refer to as shading S. The parameter S therefore comprises both the illumination falling on the object’s surface and any differences in intensity caused by the slant of the surface relative to the angle of illumination. If a single illumination direction is assumed, it is then possible to reconstruct a normal map of the object with a few assumptions on the smoothness of the surface. While assuming a single illumination direction is biologically meaningful, as the sun is usually the main source of illumination in outdoor environments, assuming uniform reflectance is rarely warranted in practice. If the image reflectance cannot be assumed to be uniform, then it is necessary to first distinguish the reflectance from the shading map (which represents variation in S across the image). This problem is referred to as intrinsic image decomposition [40]. The reflectance R and shading S of an image I are related by the image formation model [41]:


(2.1) $$\begin{array}{lcr} & I=R\times S, & \end{array}$$

which leads to an under constrained set of equations, as each pixel yields three equations, one for each colour channel, but six unknowns. The number of equations can be reduced if the shading term S is assumed to be monochromatic, but the problem remains under-constrained.

This ambiguity is dealt with in practice by regularizing the problem [42]; this can be understood from a Bayesian perspective as including prior knowledge of the underlying problem to impose soft constraints on the set of solutions [43]. The most often used such set of constraints for intrinsic image decomposition is derived from the Retinex model [44], which assumes that smooth variations in image intensity are caused by variations in S, while large variations are caused by variations in R. Such priors can be encoded in a probabilistic model by assuming a heavier tail in the distribution of the gradients of R [42,45]. Other forms of priors can also be used jointly with the Retinex constraints; for example, assuming that similarly textured image patches have the same reflectance [44] or that regions with high specularities are less saturated than regions without specularities [46].

We solve equation (2.1) by minimizing a regularized problem using a set of non-local reflectance constraints similar to those proposed by [44]. First, we convert the image in log colour-space to be able to substitute log(R)=log(I)−log(S) and solve only for log(S). We can then express the minimization problem as:


(2.2) $$S=arg_{S}\;min\;\lambda_{Rx}Rx(S)+\lambda_{Tx}Tx(S)+\lambda_{Sc}Sc(S),$$

where Rx(S) is the Retinex constraint, Tx(S) is a texture constraint assuming that patches with correlated textures have the same reflectance, and Sc(S) is a constraint to remove the ambiguity on the scale of the shading. $\lambda_{Rx}$, $\lambda_{Tx}$ and $\lambda_{Sc}$ are weights that enable us to tune the strength of each constraint. Here, we used $\lambda_{Rx}=1$, $\lambda_{Tx}=1$, so that the effect of the Retinex constraint and the texture constraint are balanced, and $\lambda_{Sc}=1000$, since the Sc constraint will impact only the small proportion of brightest pixels and has to be enforced more strongly than the other two constraints. Rx(S) is encoded as:


(2.3) $$∥\omega (I)\nabla (\text{l}\text{o}\text{g}(I)-\text{log}(S))+\nabla \text{log}(S){∥}_{2}^{2}.$$

ω(I) is 0 if the gradient of the image is larger than a threshold t, 100 otherwise. Tx(logS) is the texture constraint presented in [44]. Sc(logS) is encoded as:


(2.4) $$∥b(I)(\text{log}(S)){∥}_{2}^{2},$$

where b(I) is equal to 1 when I is at least as bright as 95% of the brightest pixel in I, or is otherwise equal to 0.

In practice solving equation (2.2) requires solving a very large linear system, which is impossible to solve directly due to the very large number of parameters. To address this limitation, we used matrices, which enable us to fit the equation in computer memory, thereby allowing us to employ an iterative solver to obtain the solution.

#### 2.3.2. Closed-form solutions for normal estimation

Once the problem of intrinsic image decomposition has been solved, the depth needs to be estimated from the R and S components of the image. This is usually done by estimating the normal map N of the image. Given a single lighting direction vector D, and assuming that the object’s surface is Lambertian or quasi-Lambertian, there is a simple equation [47] linking the shading component S and the normal:


(2.5) S=⟨D,N⟩,

which is under constrained when trying to solve for N. As for §2.3.1, to make the problem solvable, we need to apply two additional constraints to the model.

The planar direction of D can be selected as being the dominant direction of the gradient in the image, but the incident angle of the light source is ambiguous. We therefore set the incident light angle arbitrarily to be 45°. This value offers an optimal compromise between the vertical direction, which ensures all pixels are illuminated by a ray at less than 90° but erases all effects of the direction, and a horizontal light direction, which maximizes directional effects but also maximizes the number of pixels where the normal will point away from the light source. As this will have the effect of potentially tilting the reconstructed normal map, we correct for this by fitting a plane to the final height map and removing any linear trend in the data (see §2.3.3).

The first constraint is to enforce the smoothness of the normal map by assuming the gradient is close to 0:


(2.6) $$\begin{array}{lcr} & \nabla N=0. & \end{array}$$

The second constraint is to enforce that the direction of the normal is oriented against or towards the edge when there is a significant edge in the reflectance R:


(2.7) Θ(||∇R||−c)⟨S∇R,N⟩=0,

where S is a 90° rotation matrix, c is a threshold for the gradient of the reflectance and Θ is the Heaviside function.

With these additional constraints, equation (2.5) can be solved in the least square sense to recover the normal vector for each pixel in the image. Once again, this is done using sparse matrices to accommodate the large number of unknowns.

#### 2.3.3. Recovering height map from normal maps

The normal map N and the height map H are related by a simple, nonlinear equation:


(2.7) $$\begin{array}{lcr} & N=\frac{\left(-\partial H/\partial x,-\partial H/\partial y,1\right)}{∥(-\partial H\partial x,-\partial H/\partial y,1)∥}, & \end{array}$$

which relates the derivative of the height map to the normal map. This relation is easily derived from the definition of the normal for parametric three-dimensional surfaces [48]. For each pixel in the image, the gradient ∇H can thus be computed as $-\left(N_{x}/N_{z},N_{y}/N_{z}\right)$. Finding H is thus equivalent to solving the differential equations:


(2.9) $$\begin{array}{lcr} & \nabla H=-(N_{x}/N_{z},N_{y}/N_{z}), & \end{array}$$

which can be approximated via finite differences to get a large linear system of equations solvable by the computer. We consider a region of interest around the wings and set an initial condition that one pixel on the border of that region should be 0.

Since the choice of incident angle for the light direction D can influence the average slope on the wing of the moth in the height map, we fit a plane to the pixels in that region of interest. We then subtract the fitted plane such that, on average, the wing is flat and only nonlinear features in the wing reconstruction are visible. To produce the normal maps and height maps, the background was excluded by drawing a region of interest (the wing outline) by hand on each image. The final result is normalized between (0) black and (255) white for display (see Height map (H), figure 3).

#### 2.3.4. Adapting the model for predator visual systems

Animals vary in their visual sensitivities; for example, moth predators such as birds are tetrachromatic and have spectral sensitivities that extend into the ultraviolet wavelength range [49]. There are some excellent methods available for calibrating and converting images to different animal visual systems (e.g. modelling the viewer’s visual sensitivity, visual acuity and viewing distance [50,51]). Here, we test the feasibility of this approach, modelling the images for predator (bird) vision and specific viewing distances prior to input in the closed form model. Although this still results in RGB output images, neither the moth wing patterns nor the objects they may be mimicking (e.g. bark, leaves) are considered to have UV reflectance. However, if required, the closed-form models we use for intrinsic image decomposition could be generalized for more than three colour channels, and the resulting shading map S can be encoded as a grey-scale image. While there is no practical limitation that prevents us from applying the proposed methodology to images adapted to fit predators’ (e.g. birds) visual systems, the outputs should be interpreted with caution.

#### 2.3.5. Deep-learning models

A handful of neural network-based monocular depth estimation methods has been selected for our experiments. First, we consider BinsFormer [52], a state-of-the-art method based on transformers, to propose multiple plausible depth levels that are later aggregated using an attention-based module. At the time of model selection, BinsFormer was the top-performing model on popular monocular depth estimation benchmarks like NYU [53] and KITTI [54]. We also tested additional open source methods like 'From big to small’ (BTS) [39], a hierarchical model where the depth reconstructed from lower resolution images is used to guide successive reconstruction up to the image resolution, AdaBins [55], the base model upon which BinsFormer is based, and DepthFormer [56], another model combining convolution layers and transformers.

The convolution layers used in these architectures are known to mimic the basic function of animal visual systems [57]. Transformer modules on the other hand, are based on mechanisms of self-attention [58], which is in turn derived from the older Sigma-Pi units [59]. Sigma-Pi units mimic the behaviour of interneurons, which are known to play an important role in the visual cortex of animals [60]. As such, the selected monocular depth estimation models do represent a mechanical approximation of the visual system of biological brains. These machine learning-based models were run for images of all moths described in §2.2.

### 2.4. Multi-view three-dimensional reconstruction

When using multiple cameras, the distance and position of a point relative to the cameras can be computed from the relative image position of this point’s projection on the images’ planes. If the cameras are fronto-parallel with the same internal parameters, then the depth z of a point relative to the cameras can be computed from the disparity d (difference between the coordinates in pixels of the point projection):


(2.10) $$\begin{array}{lcr} & z=\frac{Bf}{d}, & \end{array}$$

where B is the distance between the cameras, referred to as the baseline, and f is the cameras’ focal length in pixels. This is also the fundamental principle that species with fronto-parallel eyes use to get a perception of depth using binocular vision [5].

When the cameras are not fronto-parallel, the images can be rectified to simulate a fronto-parallel setup [61]. The process of reconstructing the three-dimensional shape of an object by aggregating the depth measurements obtained from triangulation is often referred to as shape from motion or structure from motion [62]. Unlike other vision-based methods for depth estimation, multi-view reconstruction based on correlation can be made insensitive to perspective cues and texture gradients, even when they change across different views, using zero-mean normalized cost functions [63]. However, three-dimesional reconstruction may be more challenging in situations where uniform textures or self-repeating patterns make the identification of similar points in the images ambiguous. This weakness of multi-view reconstruction systems is the principle at the core of three-dimensional illusions such as autostereograms [64], which can recreate a perception of depth from a single flat image. While in theory, prey may evolve a camouflage pattern that uses these principles to defeat predators with binocular vision, we are not aware of any examples.

We used the state-of-the-art multi-view reconstruction package Meshroom [62] to reconstruct a three-dimensional model from images of *E. salaminia*. We chose this particular species because it has remarkable wing patterning (figure 1), and use of a preserved, rather than a trapped specimen for this part of the work, also allowed us to take multiple images from different viewpoints without the animal moving (which would make three-dimensional reconstruction impossible). Images (*n* = 31) were captured at 6000 px x 4000 px resolution using the DSLR camera described above (§2.1). Diffuse and multi-directional illumination was provided by three LED light banks (Phottix M200RGB; New York) positioned equidistant from the specimen. To optimize the reconstruction of the camera’s relative positions for the photogrammetry, we positioned small coloured circles at random positions on a whiteboard, which was placed underneath the moth specimen.

## 3. Results

### 3.1. Natural three-dimensional surfaces

**Figure 4.**
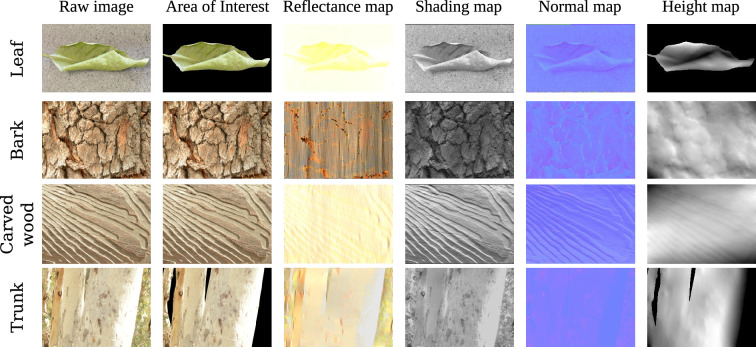
Monocular reconstruction using the closed-form solution for different natural patterns (a curled leaf, bark, carved wood and a tree trunk) that the moths may mimic. The outputs are the reflectance map ((R), shading map (S), normal map (N) and the height map (H).

**Figure 5.**
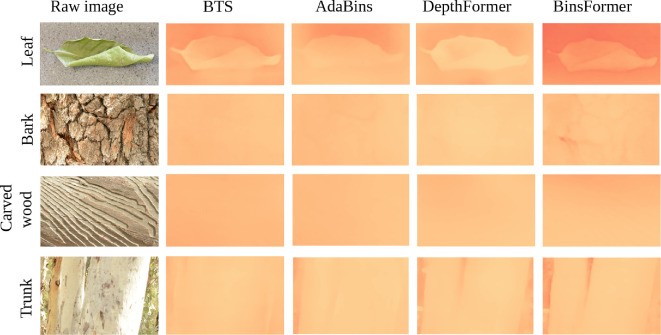
Deep learning reconstruction for different natural three-dimensional surfaces that the moths' wing patterning may mimic. The raw input images are in the left column, while subsequent columns reveal the output images (depth maps) for each algorithm.

The three-dimensional reconstruction of natural surfaces revealed large differences between the two methods (figures 4 and 5). The closed-form solution produced depth maps that were consistent with surface relief, but the outputs of the deep-learning models were more variable. The deep-learning models performed reasonably well for the images of the curled leaf and the eucalypt trunk (particularly BinsFormer), but none were able to produce depth maps for the bark or carved wood. This suggests that the deep-learning models perform better when reconstructing whole objects in visual scenes, rather than detailed texture and relief of an object that is viewed close up.

### 3.2. Shape from shading

**Figure 6.**
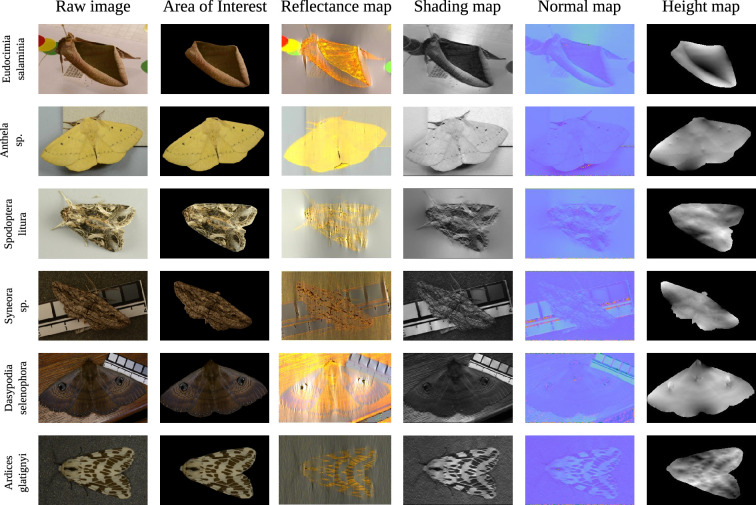
Monocular reconstruction for each species of moth using the closed-form model for shape from shading. The background is subtracted from the raw image to delineate the region of interest, which is decomposed into the reflectance map and shading map during the computations. In the normal map, the RGB channels correspond to *x,y* and *z* components of normal, which are then used to compute the final height map (normalized for display). In the height map, lighter areas of the image are closer to the viewer than darker areas.

The monocular reconstructions of the wing patterns using the closed-form solution, shown in figure 6, reveal varying amounts of depth information produced by the height maps for all species. Importantly, most of the variation in the height map for *E. salaminia* occurs in the regions of the wing (the front and rear edges) that contain illusory depth to the human observer (figure 6). As expected, other moth specimens, such as *Anthela* sp. and *D. selenophora* have relatively flat height maps, but with small bumps representing high contrast features, such as the black dots (*Anthela* sp.) or eyespots (*D. selenophora*) present on the wings. Specimens with highly textured wings such as *S. litura*, *Syneora* sp. and *A. glatignyi* yielded a height map corresponding to their wing pattern, where dark regions of the wing produce concave areas in the height map.

### 3.3. Deep learning

**Figure 7.**
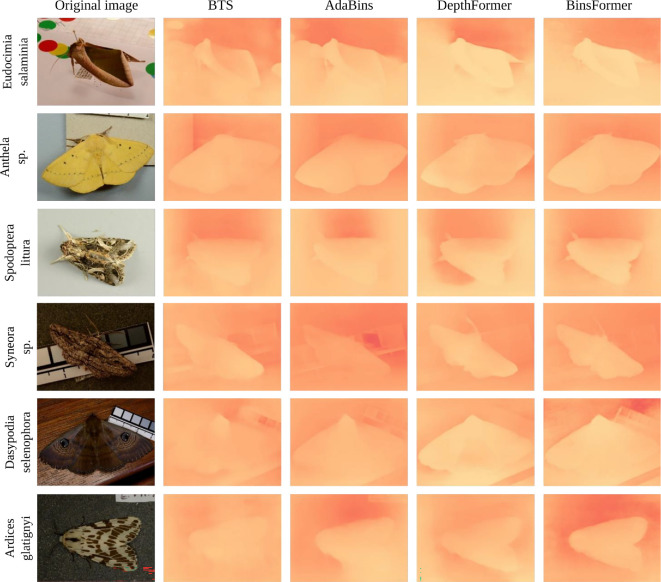
Monocular reconstruction for each species of moth using four deep-learning models. Original images for each moth species (left column) and implied depth map outputs for each monocular depth estimation (MDE) model (from left to right): BTS ('From big to small'), AdaBins, DepthFormer and BinsFormer. The relative depth estimates are encoded by colour, with red being further away and yellow being closer to the viewer.

The deep learning monocular depth estimation models revealed variation in the relative depth estimates for *E. salaminia*, with the BTS and AdaBins models producing clearer depth map outputs than the DepthFormer and BinsFormer models (figure 7: top row). In contrast to the closed-form solutions, the reconstructions for the other five species of moth resulted in no observable variation in depth across the surface of the wings (figure 7: rows 2−6).

### 3.4. Multi-view three-dimensional reconstruction

**Figure 8.**
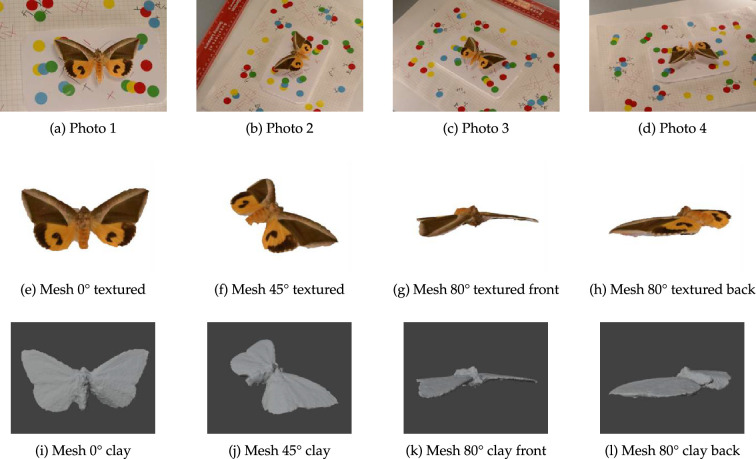
Examples of original images of *E. salaminia* (photos 1–4) taken from different view points (a–d) and used for the multi-view three-dimensional reconstruction. The outcome of these reconstructions is a textured (e–h) and clay (i–l) mesh viewed from angles of 0° (overhead: e and i), 45° (from right: f and j), 80° (from in front: g and k), and 80° (from behind: h and l).

The multi-view three-dimensional reconstruction of the wing patterns of *E. salaminia* revealed that, as expected, the surfaces of the wings are approximately flat (figure 8). Thus, the physical shape of the wing surface does not match that expected when observing the wing patterning from a single viewing point (figure 1).

### 3.5. Closed-form shape from shading adapted for bird vision

**Figure 9.**
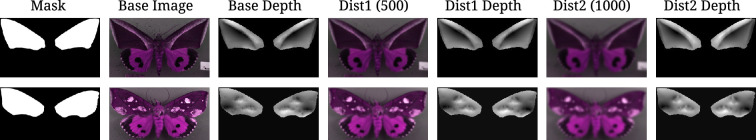
Monocular reconstruction of the wing patterns of *E. salaminia* (top row) and *E. cocalus* (bottom row) for simulated bird (blue tit) vision at viewing distances of 5 m (Dist1) and 10 m (Dist2).

We evaluated the performance of the Retinex-based model on images of the moths *E. salaminia* and *E. cocalus* calibrated for bird vision using micaToolbox [51], an Image J [65] plugin. MicaToolbox [51] was first used to model the cone catches (using the blue tit, *Cyanistes caeruleus* as a viewer), and then to simulate the pattern information for different viewing distances (based on the bird’s visual acuity) [66]. As shown in figure 9, the reconstructions match the findings we observed with the RGB images (figure 6). Correlating the pixel arrays in these images yielded correlation coefficients all *r* > 0.98 for correlations between the image illustrating base depth and the images modelled for bird vision at 5 m (Dist1) and 10 m viewing distances (Dist2) (figure 9). Modelling the images for bird vision therefore did not influence the findings; in fact, the spectral sensitivity curves of digital cameras are evenly spaced across the 400−700 nm wavelength range, which should yield reasonable reconstructions (figure 9).

## 4. Discussion

It has long been suggested that animal patterning might exploit the viewers’ mechanisms of processing depth information [11–14]. However, studying the role of depth perception in object recognition in non-human animals is challenging. In this paper, we have explored the application of computer vision methods to this problem, showing that, as for human observers, monocular depth estimation algorithms can be fooled by illusory depth cues generated by the moths’ wing patterning. While these approaches cannot replace behavioural studies with animals, this novel application of computer vision to animal camouflage may inspire future research investigating how animals process three-dimensional visual information.

### 4.1. Closed-form versus deep-learning monocular reconstruction

The closed-form approach based on the Retinex model seems to yield a good reconstruction of fine details and gives a shape reconstruction matching expectation, particularly in the case of *E. salaminia* (figure 6). With this method, the reflectance map (R) tends to be uniform in terms of intensity, meaning that shading (S) is the main contributor to the height map, causing dark patches to be reconstructed as holes and bright patches to become bumps. Thus, the Retinex model is sensitive and responds to depth in natural surfaces as well as to all types of moth wing patterns we tested. While this might be seen as a methodological limitation (many of the moth wing patterns were not expected to have depth cues; figure 2), high contrast regions are common in animal camouflage patterns and are an essential component for strategies such as disruptive colouration, where internal markings such as false edges prevent detection/recognition of the body’s true outline [67]. In this case, the high contrast false edges generated by the animal’s patterning strongly activate the edge detectors, making it difficult for predators to differentiate these false edges from the true edges corresponding to the body’s actual boundaries [68]. Although high contrast markings may serve for camouflage via a number of mechanisms [69], it is unlikely that all the moth wing patterns we tested here invoke a false impression of depth in the viewer.

The high-contrast disruptive patterns on the white tiger moth, (*A. glatignyi*) were reconstructed as patches with different depths using the closed-form reconstruction (figure 6). In human vision, the perceived brightness of an object changes with viewing distance: dark objects are typically interpreted as being further from the viewer, while lighter objects are perceived as closer [70]. The Retinex model, which is based on human vision, incorporates perceptual components of vision (colour constancy—the ability to perceive an object’s colour despite changes in illumination) with information received from the photoreceptors [71] and is one of the most widely used models for image enhancement [72] and three-dimensional scene reconstruction [73]. A future challenge is to quantify (e.g. with behavioural experiments) how the visual cortex of non-human animals solves the problem of distinguishing between an object’s reflectance (i.e. the object’s physical properties) and its shading (i.e. the illumination) and thus whether the Retinex model is appropriate for modelling animal visual systems. Of course, it is also important to note that moth predators may also use senses other than vision to detect prey (e.g. echolocation in bats [74]).

**Figure 10.**
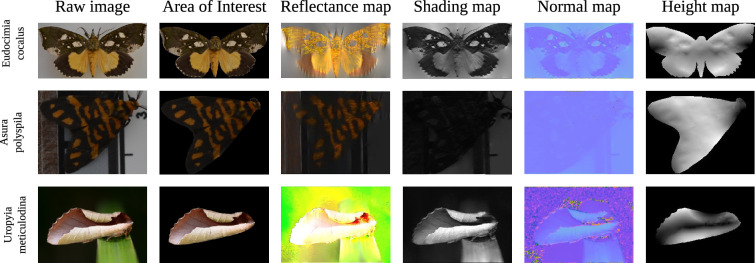
Monocular reconstruction using the Retinex closed-loop model on additional images of moths (*E. cocalus* and *A. polyspila*) with high contrast wing patterns, and patterns with strong pictorial cues (*Uropyia meticulodina*, Notodontidae). Images: *E. cocalus* and *A. polyspila*: J.L.K.; *U. meticulodina*: LiCheng Shih, Flickr.

**Figure 11.**
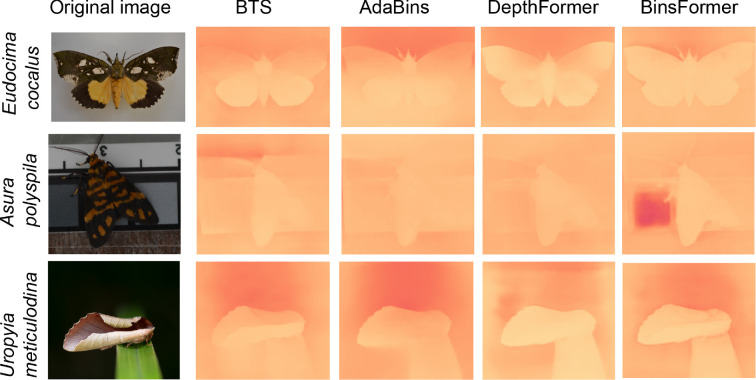
Monocular reconstruction using deep-learning models on images of the same moths as in figure 10. Depth maps were generated by BTS, AdaBins, DepthFormer and BinsFormer models, respectively. Lighter areas of the image represent areas closer to the viewer.

A limitation of the closed-form solution is that it does not encode all prior knowledge and experience that a predator uses to detect and recognize a moth in its natural environment. This problem remains when the image is modelled for predator spectral sensitivities (the wavelengths of light the eye responds to) and predator visual acuity (the ability to resolve spatial detail). As we have seen, in the case of the closed-form solution, the smallest cues of depth variation will trigger a response. Deep-learning models, which have strong priors embedded into them (e.g. a deep-learning model will learn from the training data that certain coloured objects have gradients and these gradients should not be mistaken with shading effects), can help distinguish between strong and trivial depth cues. In comparison to the closed-form solution (figures 6 and 10), the deep-learning models only reconstructed depth information for the two moth images with strong pictorial cues: *E. salaminia* and *Uropyia meticulodina* (figures 7 and 11). Both these species display a form of camouflage known as masquerade, where an animal resembles an inedible or unprofitable object [75]. Here, camouflage is achieved by avoiding recognition rather than detection; a strategy that relies on predators having previous experience with the object being mimicked (in this case, leaves) [75]. Although the deep-learning models, we used are unlikely to have had experience with leaves, the priors are both learned from the data and enforced during training, allowing generalization of objects and visual scenes. In this respect, the outcomes of the deep-learning models are promising, suggesting that some types of moth wing patterning (e.g. those that bear strong resemblance to natural objects) are more likely to provide false depth cues.

Of course, other types of defensive patterning, such as eyespots, may be effective because of their salience rather than because they create the illusion of a three-dimensional shape. For example, the eyespots of the southern old lady moth (*D. selenophora*) have high contrast internal features such as a 'pupil’ and 'specular highlights’ (figure 2). In butterflies, large eyespots can be effective in intimidating predators to reduce the risk of attack [76], without the need for any resemblance to eye shape. However, the orientation of the 'specular highlights’ (bright spots caused by reflected light) within the eyespot markings of some Lepidoptera (butterflies and moths) can be an important factor. Models with specular highlights that are consistent with overhead illumination (as would occur naturally) have significantly higher survival rates [77]. Eyespots that mimic the eyes of a predator’s enemy can also cause an aversive response in predators [78], but the role of three-dimensional shape mimicry in this context remains unexplored.

The monocular reconstructions of the closed-form solution for the wings of *E. salaminia* (figure 6) and *U. meticulodina* (figure 10) show the convex ’roll’ of an apparent leaf in a clearer way than the deep- learning models (figures 7 and 11). The outputs of the deep-learning models, particularly for species other than *E. salaminia* and *U. meticulodina*, tend to assume that the reconstructed surface is very smooth and changes slowly. This assumption is a safe one for general surfaces, but limits the ability of the model to reconstruct fine details on the wings. The tendency of depth reconstruction networks to be over-smooth when presented with samples outside of their training set has been observed in other contexts [79]. This is an important limitation of the application of deep-learning models to animal colouration, which often features fine-scale patterning such as that shown by the tobacco cutworm (*S. litura*; figure 2). It is important to note that the image inputs for the models tested here were all RGB because deep-learning algorithms were developed on the basis of human vision.

An important question when analysing the results of our reconstructions is how holes and spots are affecting the output of our algorithm. High contrast areas can be caused by sharp variations in height, and the Retinex based algorithm will predict variations in height for high contrast spots and dots (see figure 10). This is especially apparent when reconstructing elements such as bark (see figure 4). Now, it is hard to argue that darker spots on moth wings induce an illusion of depth, at least not to the extend of the patterns found in species like *Eudocimia salaminia* (even if some wings pattern could be interpreted as approximating the kind of pattern seen on bark). Spots can be recognized and identified as other elements by different visual systems. *Dasypodia selenophora* for example, displays spots on its wings that may resemble the eyes of a predator’s enemy [78], and/or may function for deterrence [80]. Nonetheless, the response of the shape from shading models when presented with a dark or bright spot shows how different patterns and strategies interconnect. This also suggests that patterns which evolved for a certain purpose (e.g. disruptive patterns that break up the wing’s outline, or spots that function for deterrence) can evolve for another purpose (i.e. inducing an illusion of depth). In contrast to the closed-loop algorithm, the deep-learning models do not appear to reconstruct high contrast areas such as spots as changes in depth. This finding is the same for the black and white patterns of *A. glatignyi*, the white wing patches in *Eudocima cocalus*, and the aposematic patterns of *Asura polyspila* (figures 7 and 11).

Overcoming the limitation of resolving fine details might require building a specific dataset to train the models, for example using natural visual backgrounds (e.g. leaves, bark) rather than the urban scenes used in NYU [53] and KITTI [54]. However, the training set would influence the results and the creation of a large enough dataset could be challenging. Another possible mitigation would be to enforce specific priors on these models to match the expected priors used by a predator’s brain. For example, a biological application of this would be to incorporate parameters that are known to contribute to predators’ search images [81]. In this context, deep-learning models have an edge over closed-form solutions, as it is easier to add more priors (embedded in the loss function used for training [43]) without over-complicating the algorithm and, hence, the computation.

The deep-learning models tended to set the bottom of the image closer than the top, which is representative of many images of visual scenes (foreground objects are at the bottom of the image), but is not the case for the moth images. This indicates that deep-learning models are strongly influenced by the prior models used in the training set, which is both encouraging as they are learning the visual characteristics of the training set, but also concerning as unwarranted priors will likely have a big effect on the model’s outcomes.

Overall, the closed-form approach and deep-learning models are complementary and model selection will depend on the user requirements. The closed-form solution provided results with intermediate steps that are more explainable and based on general theoretical principles. While deep-learning models derive their performance from priors derived during their training and that potentially may be used to model predator experience, they are sensitive to the choice of training data and, in the context used here, have difficulty reconstructing fine details.

### 4.2. Multi-view three-dimensional reconstruction

Our three-dimensional mesh model produced an approximately flat wing surface (figure 8), demonstrating that the multi-view reconstruction is unaffected by the camouflage patterns on the wings of the green fruit-piercing moth (*E. salaminia*). This is expected as motion parallax and stereopsis provide important, and additional sources of depth information to monocular cues. Indeed, there is good evidence that binocular vision plays an important part in defeating camouflage patterns that incorporate depth cues such as enhanced edges (where dark colour patches are bordered by a darker colour and light patches are bordered by a lighter colour [24]). Adams and colleagues [82] presented human observers with images of snakes with and without enhanced edge patterning, which were presented to either one eye (monocular vision) or both eyes (binocular vision). While snakes with enhanced edge patterns were slower to detect, this effect was only observed for monocular viewing conditions [82]. Any depth information generated by the patterning on the moth wings would, therefore, only be effective when viewed monocularly and/or from a fixed viewpoint; any head or body movements of the viewer would reveal the true form of the wing shape. This important point is illustrated by comparing the images of *E. salaminia*; the multi-view image reconstructions (figure 8) reveal a flat wing surface while the single image reconstructions (figures 6 and 7) reveal significant curvature at the edges of the wings.

While pictorial depth information can be defeated by binocular vision [82], monocular cues still provide important depth information to animals. Animals such as pigeons [28], chicks [29], cuttlefish [30] and macaques [31] are known to use SFS to resolve three-dimensional shapes. Furthermore, birds are able to discern the direction of illumination in an image to resolve convex and concave surfaces [29,31]. This demonstrates that animals use fundamental knowledge about the world (priors) to infer information about objects in three-dimensional visual scenes. In theory, therefore, like computer vision algorithms, animal predators should potentially be fooled by false depth cues under some viewing conditions.

## 5. Conclusions

In summary, we show that three-dimensional image reconstruction methods, which have broad applications in the field of computer vision [38], are potentially a valuable tool for understanding how animal patterning might defeat low-level visual processing; specifically, the depth information obtained using monocular cues. Furthermore, comparison of different reconstruction methods can provide insights into how different types of patterning may deceive the viewer. Our findings add to the increasing body of work using computer vision for biological applications [83], including classifying animal colour patterns [84], examining geographic variation in animal colouration [85] and investigating egg pattern signatures in birds [86]. Future work in this area would first incorporate pictures of the same species of moth photographed in different illumination conditions and on different backgrounds to further test the application of these models. The approach could then be extended to include a broader range of species with potential pictorial cues (and not just Lepidoptera), as well as considering patterning that may serve for other functions (e.g. signalling). Our computational models could be combined with empirical behavioural studies with real animals (e.g. by three-dimensional printing the reconstructed meshes) in controlled or natural settings, providing deeper insights into the effectiveness of camouflage in different contexts. Finally, our methods may be used to investigate how environmental factors, such as variation in the illumination conditions (particularly shadows), and habitat characteristics (e.g. depth cues in the visual background), influence the recovery of depth cues.

## Data Availability

Supporting codes and data are available on Zenodo at: [87].
